# Miniature Dielectric Barrier Discharge Nonthermal Plasma Induces Apoptosis in Lung Cancer Cells and Inhibits Cell Migration

**DOI:** 10.1155/2017/8058307

**Published:** 2017-01-24

**Authors:** Surya B. Karki, Eda Yildirim-Ayan, Kathryn M. Eisenmann, Halim Ayan

**Affiliations:** ^1^Department of Bioengineering, College of Engineering, University of Toledo, Toledo, OH 43606, USA; ^2^Department of Orthopaedic Surgery, University of Toledo Medical Center, Toledo, OH 43614, USA; ^3^Department of Biochemistry and Cancer Biology, University of Toledo Health Science Campus, Toledo, OH 43614, USA; ^4^Department of Mechanical, Industrial, and Manufacturing Engineering, College of Engineering, University of Toledo, Toledo, OH 43606, USA

## Abstract

Traditional cancer treatments like radiotherapy and chemotherapy have drawbacks and are not selective for killing only cancer cells. Nonthermal atmospheric pressure plasmas with dielectric barrier discharge (DBD) can be applied to living cells and tissues and have emerged as novel tools for localized cancer therapy. The purpose of this study was to investigate the different effects caused by miniature DBD (mDBD) plasma to A549 lung cancer cells. In this study, A549 lung cancer cells cultured in 12 well plates were treated with mDBD plasma for specified treatment times to assess the changes in the size of the area of cell detachment, the viability of attached or detached cells, and cell migration. Furthermore, we investigated an innovative mDBD plasma-based therapy for localized treatment of lung cancer cells through apoptotic induction. Our results indicate that plasma treatment for 120 sec causes apoptotic cell death in 35.8% of cells, while mDBD plasma treatment for 60 sec, 30 sec, or 15 sec causes apoptotic cell death in 20.5%, 14.1%, and 6.3% of the cell population, respectively. Additionally, we observed reduced A549 cell migration in response to mDBD plasma treatment. Thus, mDBD plasma system can be a viable platform for localized lung cancer therapy.

## 1. Introduction

Lung cancer is one of the most common cancers in the United States and is the leading cause of cancer-related deaths [1]. According to the American Lung Association, lung cancer mortality rates are higher than that of colon, breast, and prostate cancers combined [2]. In 2015, approximately 158,040 Americans died from lung cancer, about 27 percent of all cancer deaths [3].

Surgery, radiotherapy, and chemotherapy are conventional lung cancer treatment techniques used to combat the disease. Yet, all these techniques have some limitations [4–7]. Surgical resections commonly used to dissect the tumor may leave behind residual cancer cells. Radiotherapy involves a radiation hazard to normal tissue, while chemotherapy causes both neuropathies, poisons “healthy” cells at the vicinity of tumors, and induces side effects such as nausea, flu-like symptoms, and hair loss [6, 7]. Furthermore, all these techniques have low therapeutic efficiency. Researchers and clinicians have sought a “magic-bullet” therapy that induces apoptosis in cancer cells, while preserving the surrounding healthy cells [8, 9].

Plasma medicine is an emerging field that has investigated the application of physical plasma in cancer therapy. Nonthermal atmospheric pressure plasma has been utilized in various therapeutic applications including surface sterilization [10–12], surface modification [13], blood coagulation [14], wound healing [15], biofilm inactivation [16–18], dental treatment [19–21], and cancer therapy [22–25]. Prior research in the application of plasma medicine in cancer treatment in a subset of cancers has demonstrated promising results.

Kim et al. [1] utilized fiber-based jet plasma to induce apoptosis in lung cancer cells. Their results demonstrated that due to their small diameter and low gas flow rate, microplasma jet devices induced apoptosis but not necrosis. Weiss et al. [26] used jet plasma on prostate cancer to study the antiproliferative effect of plasma by redox and apoptotic signaling pathways. Huang et al. [5] studied the effect of a dielectric barrier discharge plasma needle on lung cancer cells. Their results demonstrated that increased applied power and prolonged exposure time improved the efficiency of apoptotic induction in cultured lung cancer cells. Keidar et al. [27] investigated the effects of plasma treatment in bladder cancer xenografts. Their results suggest that local application of nonthermal plasma selectively reduced the size of bladder cancer tumors in nude mice. Smaller tumors of ~5 mm were ablated completely after 2 min of plasma treatment, while larger ones decreased in size [28]. Colorectal, glioblastoma, and melanoma cancer cells also underwent apoptosis upon treatment with plasma in vitro [29–31].

In plasma medicine for cancer studies, so far jet plasmas (often referred as plasma needle) and dielectric barrier discharge (DBD) plasmas with large electrodes (35–80 mm diameter) have been used [5, 11, 32]. Yet, jet plasmas are associated with possible glow-to-arc discharge transitions and are less stable compared to conventional DBDs [22, 33]. Further, DBD plasmas with large electrodes create an extensive number of randomly distributed microdischarges over the dielectric surface, which limits its selective and precise targeting abilities, especially for small tumors [5, 34, 35]. To address these limitations and to target areas of interest precisely without requiring any gas flow, there is a demand for a system that can create nonthermal DBD plasma using a small electrode.

In this study, we have constructed a miniature dielectric barrier discharge (mDBD) electrode with a 6 mm diameter to treat lung adenocarcinoma epithelial cells and to selectively initiate apoptosis in a target cell population. The 6 mm electrode size was chosen to resemble bronchoscopes used in surgeries for lung biopsies. The objective of this study was to investigate whether mDBD plasma induced apoptotic cell death in treated A549 lung adenocarcinoma epithelial cells in response to varying treatment durations. mDBD plasma treatment of 120 sec and shorter initiated apoptotic death, promoted cell detachment and blocked tumor cell migration in A549 lung cancer cells. Therefore, these finding suggest further exploration and development of mDBD plasma for localized lung cancer therapy.

## 2. Materials and Methods

### 2.1. Fabrication and Characterization Method of the Miniature Dielectric Barrier Discharge Plasma System

**Figure 1:**
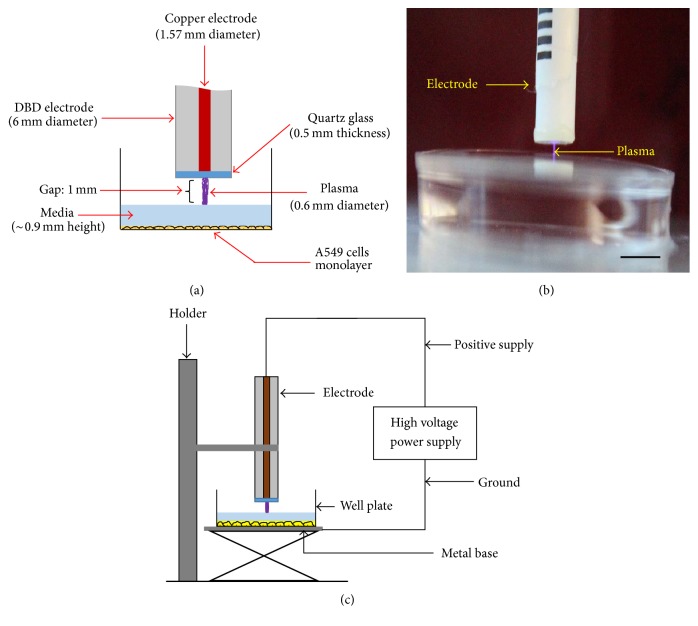
(a) Schematic of the experimental setup showing the plasma system and sample. (b) Photograph of the ignited miniature DBD plasma on the sample. (c) Schematic of the electrical setup. Scale bar is 6 mm.

The mDBD plasma system was fabricated to generate a nonthermal thin discharge channel for treating lung adenocarcinoma epithelial cells. mDBD is not a jet plasma, and therefore it does not require flow of a special working gas. It operates in atmospheric pressure ambient air. The mDBD electrode consists of a copper rod (1.57 mm diameter), which is coaxially inserted in a nonconductive polyetherimide (Ultem®) material (6 mm diameter) with a length of 400 mm (Figure 1). The flat tip of the copper rod was covered with clear 0.5 mm thick fused quartz, acting as a dielectric barrier (Figure 1(a)). The electrode system was powered by a pulsed power supply (Advanced Plasma Solutions) that can generate up to 35 kV peak voltage with a 100 Hz–1 kHz repetition rate. In this study, nonthermal plasma was generated with 12 kV at 1000 Hz with a 10 *μ*s pulse width.

The electrode was placed 1 mm above the cell culture medium covering the A549 cells (Figure 1(a)). Upon application of high voltage, a visible mDBD plasma discharge was produced between the quartz and the surface of the cell culture medium (Figure 1(b)). mDBD plasma discharge was applied to the cell culture medium based on predefined treatment time.

The high voltage and current waveforms were acquired using a wide bandwidth voltage probe (PVM-4 130 MHZ 1000 : 1 <5% error, North Star High Voltage) connected in parallel with the plasma electrode. The high voltage cable was passed through a magnetic core Pearson™ current monitor (1 V/A, ±1% sensitivity, 1.5 ns useable rise time) to record the waveform of the discharge current. Signals from the current and voltage probes were recorded by a digital storage oscilloscope (TDS 2014C, 100 MHz, 2 GS/s, Tektronix, USA).

### 2.2. Treatment Method of Lung Adenocarcinoma Epithelial Cells with mDBD

In this study, A549 lung adenocarcinoma epithelial cells (ATCC, VA, USA) were treated with mDBD plasma for various times. Cells were incubated in Kaighn's modification of Ham's F-12 medium (F-12K medium) (ATCC) supplemented with 10% fetal bovine serum (FBS) (ATCC) at 37°C and 5% CO_2_ with humidity. Once confluent, the cells were detached, seeded into 12-well plates at seeding density of 1.5 × 10^5^ cells/well, and incubated overnight at 37°C.

On the day of plasma treatment, A549 cells were washed with phosphate-buffered saline (PBS) and 340 *μ*L of fresh growth liquid medium was added to the wells to create ~0.9 mm height liquid layer covering the cells during plasma treatment. Cells were exposed to the mDBD plasma for 0 (control), 15, 30, 60, and 120 sec in the presence of cell culture medium. The temperature of the medium was also measured using a Digi-Sense® thermometer (Cole-Parmer, USA) for all treatment groups. Plasma was applied to the surface of the culture medium that covered cells. The gap between the bottom side of the electrode and the surface of liquid medium was kept at 1 mm for all samples.

### 2.3. Study of the Effects of mDBD Plasma Treatment Time on Size of Detached Cell-Area

To understand whether the plasma can detach cells and whether the area of cell detachment increases with prolonged plasma treatment time, immediately after mDBD plasma treatment (day 0), cells were washed with PBS. Here, it should be noted that sample preparation takes about 1 hour, and the results obtained after plasma treatment and necessary preparation were considered as “immediately after.” Multiple zones of clearance were imaged using an inverted optical microscope with a 4x objective lens (Olympus). Images were stitched together using Photoshop CS6 software (Adobe Photoshop, CA, USA) to visualize the entire mDBD plasma treatment area as a single image. The diameter and area of the plasma-induced clearance zone were measured by using Image J (NIH) software. Cells were also stained with a live/dead assay (Life Technologies) to visualize the different zones of treated area using a fluorescence microscope (AmScope, CA, USA) with a 4x objective lens.

### 2.4. Study of the Effect of mDBD Plasma Treatment Time on Cell Viability

A live/dead assay was performed on the A549 cells 24 h after the plasma treatment to assess the effect of mDBD plasma treatment on cell viability. Cells plated on 12-well plates were treated with plasma for 0 sec (control), 15, 30, 60, and 120 sec and stained with a mixture of 4 *μ*L of calcein-AM-fluorescence isothiocyanate (FITC) and 8 *μ*L ethidium homodimer-1 (Life Technologies, OR, USA) added to 10 mL PBS. Samples were incubated for 20 min, washed with PBS twice, and then visualized under a fluorescence microscope (AmScope, CA, USA) with a 4x objective lens. Live cells stained green with calcein-AM (495/525 nm excitation/emission wavelengths), indicating intracellular esterase activity of cells. Dead cells stained red with ethidium homodimer-1 (540/635 nm excitation/emission wavelengths), indicating loss of cell membrane integrity [36–38]. Images were captured and analyzed using ISCapture image acquisition software (Amscope, CA, USA).

### 2.5. Study of the Effect of mDBD Plasma Treatment Time on Viability of Detached and Attached Cells

**Figure 2:**
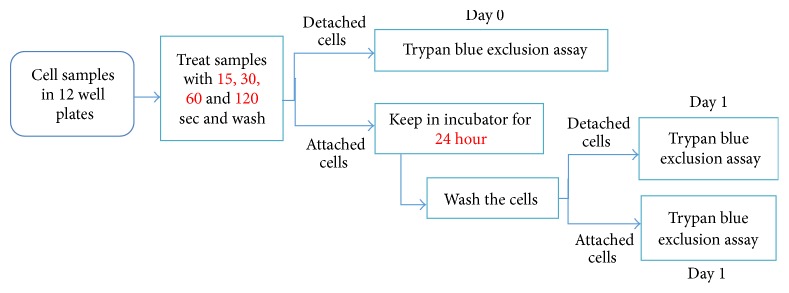
Block diagram of the experimental workflow to assess the effect of mDBD plasma treatment on the viability of detached and attached A549 lung cancer cells immediately after plasma treatment (and after 1 hour of sample preparation) and 24 h later.

To evaluate cell viability in both attached and detached cells, on day 0 immediately after mDBD plasma treatment (and after 1 hour of sample preparation) cells were washed with PBS to collect detached cells. Detached cells were counted with a hemocytometer using a trypan blue exclusion assay to evaluate cell viability. The remaining attached cells were immediately covered with culture medium and incubated for 24 h to observe the post plasma effect upon cell viability. After 24 h (day 1), cells were washed with PBS twice to collect the detached cells and trypan blue exclusion was used again to count live and dead cells. Cells attached to the wells were then trypsin released, and cells were evaluated using trypan blue exclusion (Figure 2). Samples were prepared for each treatment time and the experiment was performed in triplicate.

### 2.6. Study of the Effect of mDBD Plasma Treatment Time on Apoptosis

Apoptosis was detected using an Annexin V-FITC apoptosis detection kit (Life Technologies) per the manufacturer's specifications. Cells (1.5 × 10^5^ cells/well) in 12-well plates were treated with plasma and incubated at 37°C for 24 h. After 24 h, cells were collected, centrifuged and resuspended in 100 *μ*L binding buffer. Propidium iodide (PI) (3 *μ*L) and Annexin V-FITC (6 *μ*L) were added to each 100 *μ*L sample and incubated for 15 min at room temperature in the dark. After incubation, 400 *μ*L of binding buffer was added to each sample, and the samples were analyzed by flow cytometry using a Becton-Dickinson fluorescence-activated cell sorter BD FACS (BD Bioscience San Jose, USA) with 488 nm excitation from the argon ion laser at 15 mW. Forward scatter threshold was set to exclude small debris. Annexin V-FITC was captured on the FL-1 channel equipped with a 631 nm wavelength filter and 52 nm bandwidth in linear mode. PI fluorescence was determined with a 557 nm filter and 48 nm bandwidth in linear mode. Data acquisition used CellQuest software (BD Bioscience San Jose, USA). At least ten thousand events were acquired per sample. Triplicate samples were run per treatment time, and each experiment was repeated four times.

### 2.7. Study of the Effect of mDBD Plasma Treatment Time on Cell Migration

A549 cancer cells on 12-well plates were exposed to the plasma for 15 sec in medium. For controls, 2 mm diameter polycarbonate inserts were placed into the 12-well plates, and cells were plated around them. The next day, inserts were carefully removed, and clearance zone was formed. Cellular debris was removed by washing with PBS. Cell migration was documented on days 0, 1, 2, and 3 using an optical microscope (Olympus, PA, USA) with a 4x objective lens (NA 0.10; WD 17 mm). Migration of cells towards the clearance zone (“covering”) was studied by measuring changes in the area of clearance zone using Image J software.

To evaluate the effects of plasma on the surface of 12-well tissue culture plates, media alone (no cells) were placed in 12-grid well plates. Well plates were then treated for 0 (control), 15, and 120 sec, and images of the culture dish bottom surface were taken using optical microscope (Olympus, PA, USA) using a 4x objective lens. After plasma treatment, cells were then added to the well plates and were kept in an incubator for days 1, 2, and 3. Images were taken on each day to observe the cell attachment and growth on the treated surfaces. Gridded 12-well plates were used to properly locate the plasma treated surface. Image J (NIH) software was used to count the cells.

### 2.8. Statistical Analysis

Each experiment was performed in triplicate and repeated at least three times. Student's unpaired *t*-test was used to access the differences between control and plasma treated groups. *p* < 0.05 was considered statistically significant.

## 3. Results

### 3.1. mDBD Plasma System Characterization

**Figure 3:**
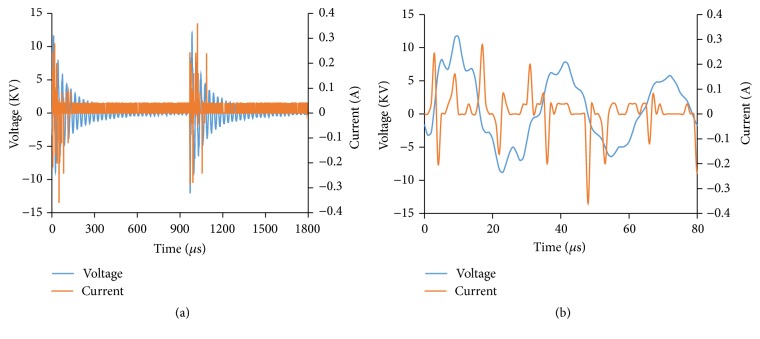
(a) Two complete cycles of voltage and current waveforms of mDBD nonthermal atmospheric pressure plasma system based on 1000 Hz frequency and 10 *μ*s voltage half-height width. (b) A close-up view of the voltage and current waveforms.

The waveforms of applied voltage and discharge current of mDBD plasma system were recorded (Figure 3). A close-up view oscillogram of a few voltage peaks is presented in Figure 3(b).

### 3.2. Change in Size of Detached Cell-Area with mDBD Plasma Treatment Time

**Figure 4:**
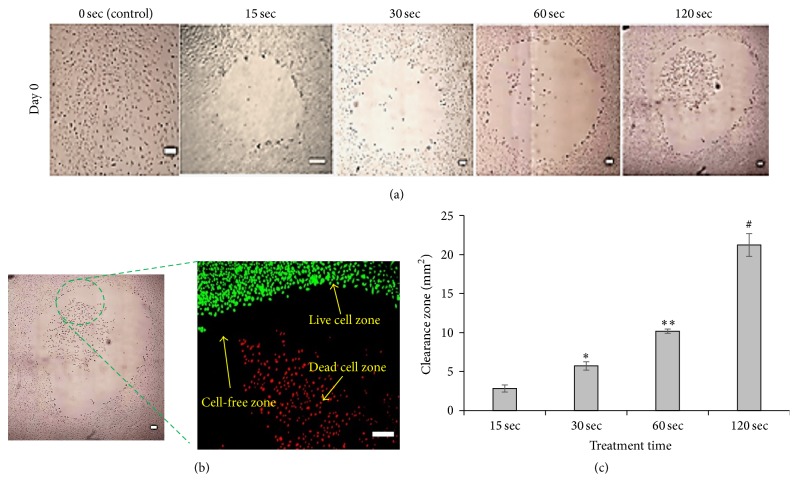
Effect of plasma on detachment of lung cancer cells. (a) Cells were treated for 15, 30, 60, and 120 sec. Except for the control, all images are stitched images of multiple pictures. (b) Zoomed image of a portion of plates treated for 120 sec showing the formation of three zones. (c) The clearance zone area expressed as a function of treatment time. Scale bars are 200 *μ*m. Data are means of the area (mm^2^) ± SD of three independent experiments run in triplicate. *∗*, *∗∗*, and # denote statistically significant differences for 15, 30, 60, and 120 sec treatments, respectively (*p* < 0.05).

Plasma treatment for 15 and 30 sec created the two zones (a cell-free and a live zone). With 15 and 30 sec of treatment, all cells detach from the treatment area and form a cell-free zone. A live cell zone surrounded the cell-free zone. Plasma treatment for 60 and 120 sec created three zones, a dead, a cell-free, and a live zone, from the center most, radiating outward (Figure 4(b)). The center of the treatment area was called the dead zone and contained apparent dead cells. A cell-free zone formed between live zone and the dead cell zone. A live cell zone formed around the cell-free zone. The combined cell-free and dead cell zones formed the clearance zone. The diameter of the clearance zone was measured using Image J. The average clearance zone diameters for 15, 30, 60, and 120 sec were found to be 1.7, 2.6, 3.8, and 5.9 mm, respectively. Using the diameter, the area of clearance zone was also measured using Image J.  Figure 4 demonstrated that with increased plasma treatment time, the area of these zones was also increasing.

### 3.3. Effect of mDBD Plasma on Viability of Lung Adenocarcinoma Epithelial Cells

**Figure 5:**
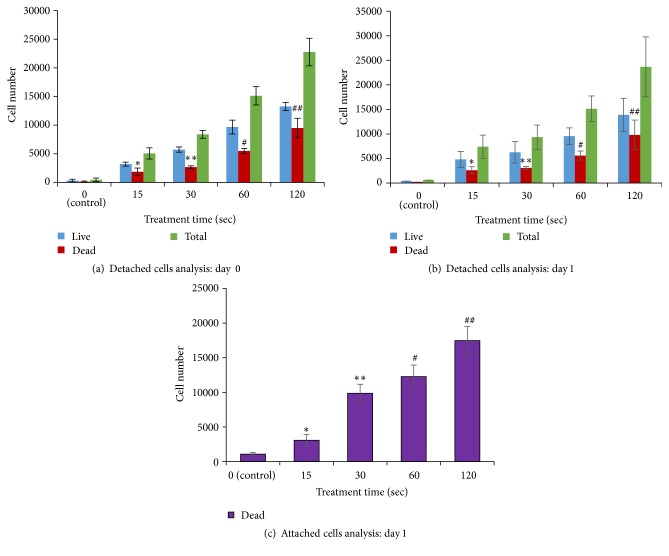
Live and dead cell count for treatment times 15, 30, 60, and 120 sec. (a) Detached live and dead cells immediately after the plasma treatment (day 0). (b) Detached live and dead cells 24 h after the plasma treatment (day 1). (c) Total number of attached dead cells on day 1. Data are expressed as mean of cell number ± SD (*n* = 3). *∗*, *∗∗*, and #, ## denote statistically significant differences between dead cells of control and 15, 30, 60, and 120 sec plasma treatments, respectively (*p* < 0.05).

This experiment was performed to observe the viability of detached lung cancer cells immediately after plasma treatment (and after 1 hour of sample preparation) (day 0) and attached as well as detached cells 24 h (day 1) after the plasma treatment. The numbers of viable and dead cells were evaluated by using a trypan blue exclusion method. We observed both viable and dead cells with the detached cell population. Viable cells outnumbered dead cells within this population on day 0 (Figure 5(a)). Detached cells on day 1 also had more live cells than dead cells for all treatment groups (Figure 5(b)). On days 0 and day 1, the numbers of dead, detached cells increased with prolonged treatment. For attached cells at 24 h after plasma treatment, the relationship of treatment time to cell death was similar to that for detached cells (Figure 5(c)). For all treatment times, attached dead cells outnumbered detached dead cells on days 0 and 1.

**Figure 6:**
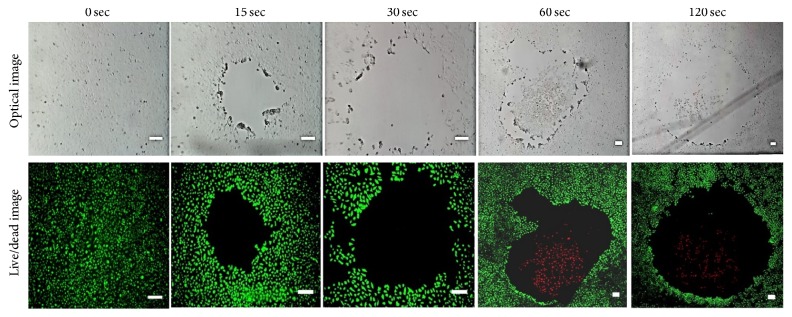
Optical and fluorescence stitched images 24 h after plasma treatment for 15, 30, 60, and 120 sec to determine cell viability. The stitched images visualize the whole treated area. Control images were taken before plasma treatment. Green is live cells. Red is dead cells. Scale bars are 200 *μ*m.

Furthermore, after counting the viable and dead cells using trypan blue, a live/dead cell assay was performed to confirm lung cancer cell death 24 h after the plasma treatment. To visualize the entire treated area, several bright fields and fluorescence images were taken of the same sample and then these images were stitched together (Figure 6). After plasma treatment, some cells detached from the surface of the well plate. With 15 and 30 sec of treatment, all cells detached from the central area. However, for 60 and 120 sec of plasma treatment, dead cells (red) remained in the treated area 24 h after the plasma treatment.

### 3.4. mDBD Plasma-Induced Apoptosis in Lung Adenocarcinoma Epithelial Cells

**Figure 7:**
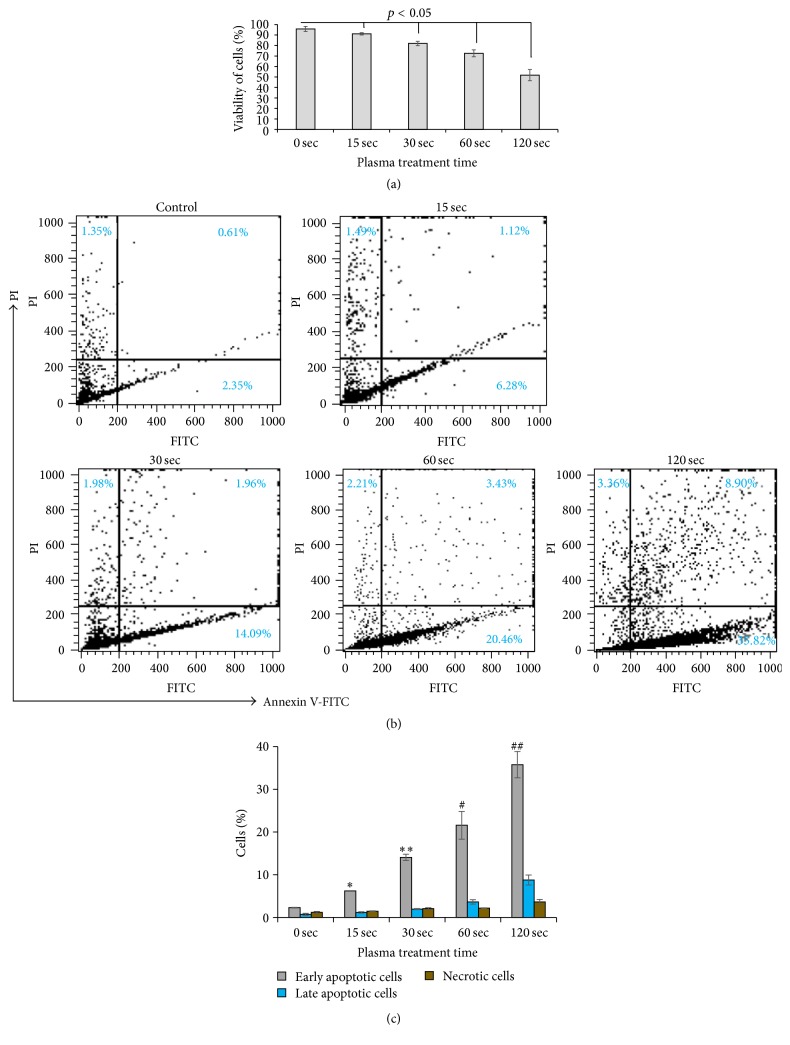
Viability and apoptosis analysis of 0 (no treatment), 15, 30, 60, and 120 sec samples using Annexin V-FITC and PI after 24 h of plasma treatment. (a) Viability percentage of cancer cells treated with plasma for four different times after 24 h of plasma treatment. (b) The percentage of cells in the four different quadrants. Upper-left: PI^+^ and Annexin V^−^, necrotic cells; upper-right: PI^+^ and Annexin V^+^, late apoptotic cells; lower-right: Annexin V^+^ and PI^−^, apoptotic cells; lower-left: PI^−^ and Annexin V^−^, live cells. (c) Quantitative depiction of cell sorting data. Data are expressed as mean of cell number ± SD (*n* = 3). *∗*, *∗∗*, #, and ## denote statistically significant differences between control and 15, 30, 60, and 120 sec plasma treatments, respectively (*p* < 0.05).

To study the apoptotic effect of plasma along with the viability of lung cancer cells after 24 h of plasma treatment, cell viability was measured using flow cytometry for all treatment time groups using Annexin V-FITC and PI. The time-dependent effect of plasma on apoptosis was observed (Figure 7(a)). The cell viability was decreased because plasma induced apoptosis or necrosis with increased mDBD plasma treatment time. The control sample was 96% viable. Upon plasma treatment for 15 sec, the viability decreased to 92%. As treatment time increased from 30 to 120 sec, the percentage of live cells decreased from 82% to 52%, respectively. For treated samples, the decrease in the percentage of viable cells was found to be linearly proportional with plasma dose (i.e., treatment time).

The mode of cell death, including apoptosis and necrosis, was further analyzed. Cells were analyzed using flow cytometry and the apoptotic effect of mDBD plasma was assessed after 24 h of plasma treatment. Annexin V-FITC and PI were used to detect apoptosis in treated cells.  Figure 7(b) represents the apoptotic stages in lung cancer cells induced by plasma treatment. The upper-left quadrant represents necrotic cells (Annexin V^−^, PI^+^), the upper-right quadrant represents late apoptotic cells (Annexin V^+^, PI^+^), the lower-left quadrant represents live cells (Annexin V^−^, PI^−^), and the lower-right quadrant represent early apoptotic cells (Annexin V^+^, PI^−^) [39]. Plasma induced an early apoptotic response in all treatment groups. The percentage of early apoptotic cells was increased with prolonged plasma treatment time. While the percentage of early apoptosis cells treated for 15 sec mDBD plasma was 6.3%, this percentage was 35.8% for 120 sec plasma treated samples. As the treatment time increased from 15 sec to 120 sec, the percentage of late apoptotic cells also increased (Figure 7(c)). For 120 sec, the percentage of late apoptotic cells was 8.9%, higher than those in the 60 sec plasma treated sample (3.4%). The percentage of early apoptotic cells was higher than the percentage of late apoptotic and necrotic cells for all treatment groups. The percentage of necrotic cells increased up to only 4% with the increased mDBD plasma treatment time from 15 sec to 120 sec. Thus, plasma elicited an apoptotic response in a significant percentage of cells in all treatment groups.

Moreover, our flow cytometry results also indicated that the plasma affects cells at the distant area from the treatment site. For the shortest treatment time (15 sec), the clearance zone had diameter of 1.7 mm; however, our flow cytometry results (Figure 7(c)) indicated that a total of 8.9% of the cells were affected by plasma, which was equivalent to a circular area with a diameter of 6.4 mm on a 12-well plate (diameter = 22 mm).

Similarly, such significant results were observed in longer treatment durations. In 120 sec treatments, the clearance zone area had a diameter of 5.9 mm. The percentage of all affected cells was 48.1%, which was equivalent to a 15.2 mm diameter circular area on a 12-well plate. Therefore, these results indicated that the plasma had an immediate effect to a certain area of cells, but also distally affects an area that was larger than the immediate treatment region.

### 3.5. mDBD Plasma Suppresses Cell Migration

**Figure 8:**
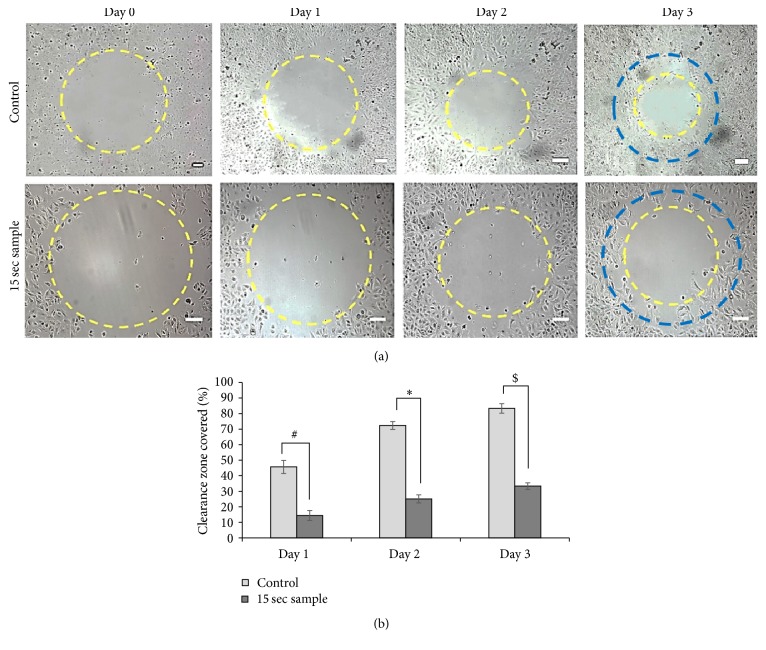
Cell migration is inhibited by plasma. (a) Control and 15 sec treated samples on days 0, 1, 2, and 3. Yellow circles on both 15 sec and control samples mark the clearance zone on each day. Blue circles on day 3 represent the initial day 0 clearance zone. (b) Normalized cumulative cell coverage based on initial clearance zone on day 0. Control images are stitched. Scale bars are 200 *μ*m. Data are the mean percentage of area covered ± SD of three independent experiments run in triplicate. #, *∗*, and $ denote statistically significant differences between control and 15 sec at days 1, 2, and 3, respectively (*p* < 0.02).

Cell migration was measured to determine the effect of plasma on cell movement. Cells were treated with plasma for 15 sec and formed an approximately 1.7 mm diameter clearance zone. For the control sample (untreated cells), an approximately 1.7 mm diameter clearance zone was created by using polycarbonate inserts to block cell attachment. Treated and untreated cells were imaged at days 0, 1, 2, and 3. Cells migrated into the clearance zone in both treated and control samples between days 0 and day 3. Migration into the clearance zone was diminished in the 15 sec plasma treatment samples compared to control samples (Figure 8(a)). Therefore, the clearance zone closed or covered via migrating cells by the 15 sec treated sample was less than the control sample on each day (Figure 8(a)). On day 1, nearly 45% of the clearance zone was covered in the control samples, while clearance zone coverage was only 12% for the 15 sec plasma treated sample. On day 3, 85% of the clearance zone was covered for control samples while coverage was only 35% for plasma treated samples (Figure 8(b)). On each day, the percentage of clearance zone covered in the control cells was higher than that in cells treated with plasma for 15 sec. These results reveal that mDBD plasma plays an important role in inhibiting the migration of A549 lung cancer cells.

## 4. Discussion

Recent progress in plasma medicine has received great attention from researchers from various fields. Specifically, nonthermal plasmas operating in atmospheric pressure have been utilized in many applications including dental treatment, sterilization of medical devices, biopolymer treatment, bacterial inactivation, and cancer treatment [40, 41]. Studies conducted with different types of DBD plasmas and jet plasmas have shown that nonthermal plasmas induce apoptosis in various cancer cells [35, 42, 43]. In this study, we created a 6 mm diameter miniature DBD electrode to generate a thin discharge channel that, when delivered to A549 lung cancer cells, induces apoptosis and halts cell migration.

The temperature of the medium was measured for all treatment groups. The results indicated that, even for our longest treatment time, the temperature did not exceed 26°C. Even after 120 sec of treatment, the temperature increased by only 1.2°C, suggesting that the temperature increase in medium does not have a thermal effect upon cells [44–46].

A549 lung cancer cells were treated with mDBD plasma for various treatment times. Due to plasma exposure, cells not only detach from each other, but also lose contact with the well plate surface [47]. mDBD plasma may induce detachment through production of reactive oxygen and nitrogen species that diffuse within the medium to be delivered to the cells. Different plasma produced species undergo interactions with living cells and induce intracellular responses including cell detachment and apoptosis [43, 47–50]. Cell detachment may be caused by the interaction of reactive plasma produced species with the cell membrane in where the reactive oxygen species may denature cell adhesion molecules [51].

Plasma treatment for 15 sec and 30 sec created two zones upon cells cultured as confluent monolayers: a cell-free and a live zone. With 15 and 30 sec of treatment, all cells detached from the central area, and no dead cells were found. In this zone, it is possible that plasma-induced reactive oxygen species disrupt adhesion molecules such as integrin and thus weaken the attachment of cells to the well plate surface, forming a cell-free zone. The live zone appears radially around the cell-free zone. In the live zone, there is no detachment or cell death. This may be explained by reduced exposure of cells to reactive species with increased distance from the treatment area [52–55]. Plasma treatment for 60 sec and 120 sec created three zones: a dead, a cell-free, and a live zone. With 60 and 120 sec treatment time, the dead zone formation may be attributed to a higher concentration of reactive species in the treatment area, which caused rapid necrosis of cells without disrupting adhesion molecules. The formation of three different zones may be a result of a diffusion gradient of reactive oxygen species concentration relative to the distance from the treatment area. Similar results were presented previously [50, 55].  Figure 4 demonstrated that the size of this zone changed with prolonged plasma treatment time. With increased treatment time, reactive species densities and their outward diffusion increased, resulting in a larger clearance zone [8].

Exposure of lung cancer cells to the plasma for all treatment groups caused cell detachment. Yet, the viability status of these detached cells was not clear. Thus, we analyzed attached and detached cells separately. On day 0, immediately after mDBD plasma treatment (and after 1 hour of sample preparation), 40% of detached cells were dead in all experimental groups (Figure 5(a)) suggesting cell death immediately after plasma treatment. For detached cells, both at day 0 and day 1, the number of dead cells increased as a function of treatment time. Although we removed all detached cells on day 0 after the plasma treatment, after 24 h later there were more dead cells in both detached and attached cells. We hypothesize that immediately after the plasma treatment, cells were dying due to necrosis. On day 1, cells might be dying because of apoptosis.

In addition to detachment, the viability of cells decreases due to apoptotic cell death induced by mDBD plasma. This is supported by the fact that nonthermal plasma can induce apoptosis in a dose-dependent manner and this might be due to DNA damage caused by the generation of reactive species such as reactive oxygen species (ROS), for example, H_2_O_2_, O_3_, OH, and reactive nitrogen species (RNS), that can pass through the cell membrane [31, 54, 56, 57]. With increased mDBD plasma treatment time, there was a decrease in lung cancer cells viability (Figure 7(a)). There were a higher percentage of apoptotic cells for all treatment groups while the percentage of necrotic cells was very low in all treatment groups (Figure 7(c)). This can be attributed to the fact that plasma does not cause instant disruption of cells. Rather plasma increases intracellular reactive species that trigger apoptosis [47, 58]. Observed differences between our results and those of others with respect to the rate of apoptosis per unit of treatment time may arise from different initial experimental conditions like electrode size, power supply, and so forth [8, 43, 59].

Plasma also significantly inhibits the migration of lung cancer cells and may ultimately induce antitumorigenic activity [57]. On the 1st day of incubation, 15 sec plasma treated cells covered 12% of its initial (day 0) clearance zone area, but control sample cells covered 45% of the initial clearance zone area. On the 3rd day, 15 sec plasma treated cells covered only 35% of the initial clearance zone area, whereas control cells covered 85% of the initial clearance zone area. The coverage of the cell-free area in the control sample (0 sec) was almost 3 times more than the plasma treated sample (Figure 8(b)). Boundary propagation in radial direction can also be presented in linear speed. From day 0 to day 3, the average radial boundary propagation for control samples was 134.7 *μ*m/day and 91.7 *μ*m/day for 15 sec treated samples. These results can be attributed to the possible changes in cancer cells surface receptors functions such as integrin-mediated cell adhesion and migration due to the reactive oxygen species generated in the plasma influenced medium [54, 56].

Plasma treatment may play an important role in cancer therapy by inducing cell apoptosis in a defined space [59]. Cell death in response to plasma treatment is correlated with all aspects of the treatment condition, specifically plasma treatment time. Yet, utilization of atmospheric pressure plasma in cancer cell treatment can be limited by the size of electrode and penetration depth of the generated plasma discharges. The feasibility of using plasma for superficial cancer has been demonstrated [56, 60]. However, our technique with a miniature DBD electrode generating a thin discharge channel is minimally invasive and more selective for cancer cells residing within a defined space. Thus, it can be a viable treatment modality for targeted lung cancer treatment therapy. This technology is adaptable to the endoscopic application due to its small electrode size and its ability to reach anatomically difficult areas unlike in conventional surgical and radiotherapy approaches.

## 5. Conclusion

Nonthermal atmospheric pressure plasma ignited from the 6 mm diameter electrode caused lung cancer cells to detach from the surface of the tissue culture plate leading to a clearance zone in the treated area of the plate. Plasma inhibits the migration of lung cancer cells. This mDBD plasma has shown promising results in inducing apoptosis in A549 cell line in vitro. Therefore, our use of this mDBD electrode may be an innovative method warranting further investigation for endoscopic treatment of lung cancer cells inside the body.

## Supplementary Material

Supplementary Fig 1A shows the plasma treated bottom surface of well plates before adding cells at day 0 (after plasma treatment) and after adding cells at day 1, 2, and 3 for 0 (control), 15 and 120 sec treated samples. Once cells were added to the plasma treated well plates, cells started to grow on the treated surface for both lowest treatment time (15 sec) and highest treatment time (120 sec) similar to the control samples. The cell number increased each day. There were more cells on day 3 than day 1 for all treated samples (Fig 1B). Thus, inhibition of migration of lung cancer cells is not due to any surface modification caused by plasma on the bottom surface of the well plate but due to the effect of plasma on lung cancer cells. Supplementary Fig 1: Cell growth occurring on plasma treated bottom surface of well plate for 0, 15, and 120 sec treated samples (A) Plasma treated surface of well plates before adding cells at day 0 and cell growth through 3 days on the plasma treated surface of well plates. (B) The increase in cell number for 15 and 120 sec treated samples was similar to the 0 sec treated sample on day 1, 2, and 3. Black lines in pictures are grid line of well plates showing that the images were taken at the same plasma treated spot at day 0, 1, 2, and 3. A yellow rectangular area of 0.64 mm² was drawn to all images to count the cells at the center of the treated area. Scale bar = 200 *µ*m. Data are the mean percentage of area covered ± SD of three independent experiments run in triplicate. ∗, ∗∗, # denote statistically significant differences between day 1, 2, and 3 for 0, 15, and 120 sec, respectively (*p* < 0.02).
