# Supplementary figures and images for: Miniature Dielectric Barrier Discharge Nonthermal Plasma Induces Apoptosis in Lung Cancer Cells and Inhibits Cell Migration
Source: Biomed Res Int. 2017 Jan 24;2017:8058307. doi: 10.1155/2017/8058307 (PMC5294360; doi:10.1155/2017/8058307)

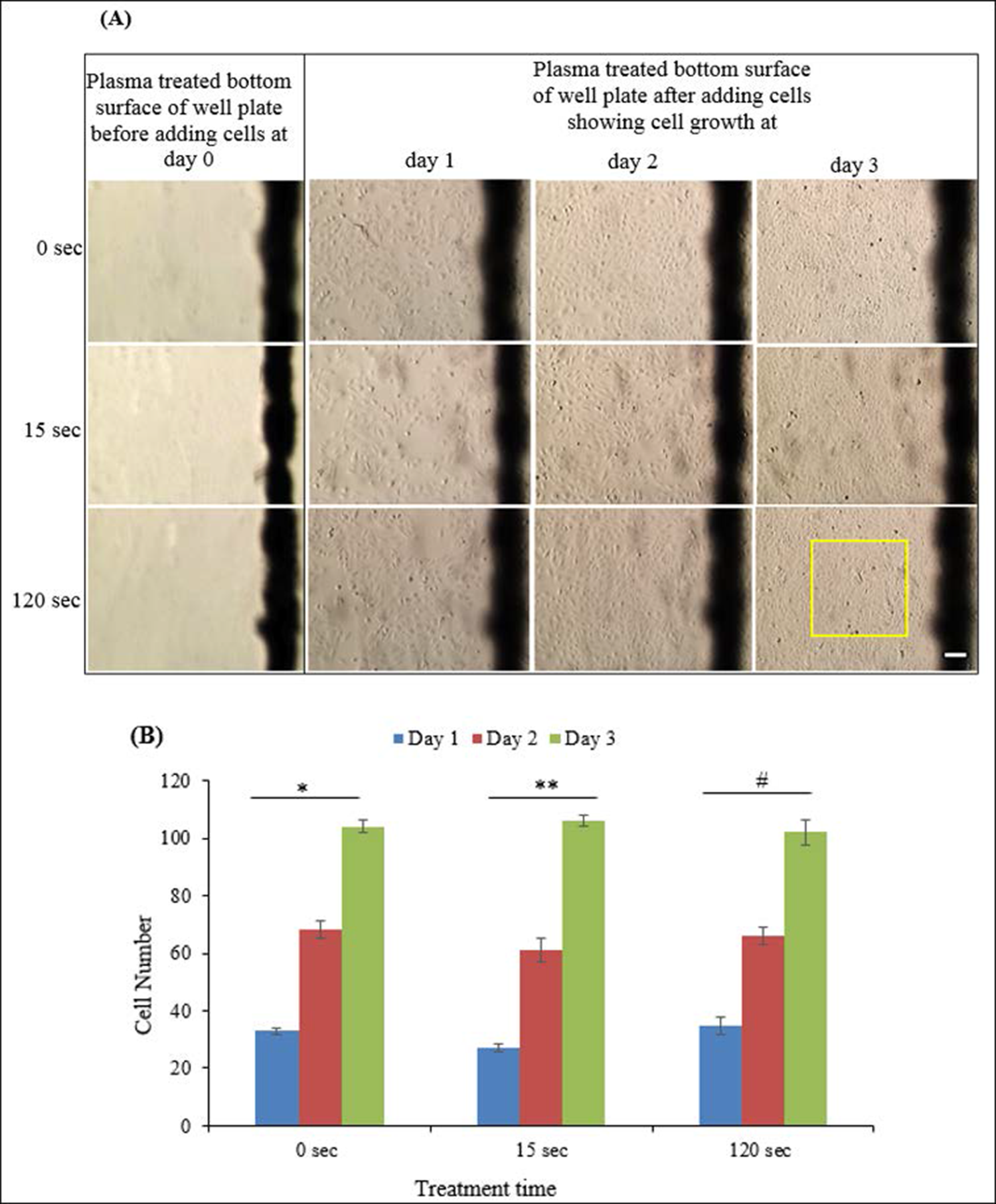

Supplement: Supplementary file 1 — Supplementary Fig 1A shows the plasma treated bottom surface of well plates before adding cells at day 0 (after plasma treatment) and after adding cells at day 1, 2, and 3 for 0 (control), 15 and 120 sec treated samples. Once cells were added to the plasma treated well plates, cells started to grow on the treated surface for both lowest treatment time (15 sec) and highest treatment time (120 sec) similar to the control samples. The cell number increased each day. There were more cells on day 3 than day 1 for all treated samples (Fig 1B). Thus, inhibition of migration of lung cancer cells is not due to any surface modification caused by plasma on the bottom surface of the well plate but due to the effect of plasma on lung cancer cells. Supplementary Fig 1: Cell growth occurring on plasma treated bottom surface of well plate for 0, 15, and 120 sec treated samples (A) Plasma treated surface of well plates before adding cells at day 0 and cell growth through 3 days on the plasma treated surface of well plates. (B) The increase in cell number for 15 and 120 sec treated samples was similar to the 0 sec treated sample on day 1, 2, and 3. Black lines in pictures are grid line of well plates showing that the images were taken at the same plasma treated spot at day 0, 1, 2, and 3. A yellow rectangular area of 0.64 mm² was drawn to all images to count the cells at the center of the treated area. Scale bar = 200 µm. Data are the mean percentage of area covered ± SD of three independent experiments run in triplicate. ∗, ∗∗, # denote statistically significant differences between day 1, 2, and 3 for 0, 15, and 120 sec, respectively (p < 0.02). [file 8058307.f1.tif]
